# Burned in Pursuit of Beauty: Injuries From Cosmetic Use of Non-Ionizing Radiation and Associated Regulatory Gaps

**DOI:** 10.1007/s11673-025-10475-y

**Published:** 2025-09-24

**Authors:** Zoe Thomas, Janneke Berecki-Gisolf, Genevieve Grant, Ken K. Karipidis

**Affiliations:** 1 Monash University Accident Research Centre (MUARC), 21 Alliance Ln, Clayton, VIC 3800 Australia; 2 Victorian Injury Surveillance Unit, MUARC, Clayton, VIC 3800 Australia; 3 Faculty of Law, Monash University, Clayton, VIC 3800 Australia; 4 Australian Radiation Protection and Nuclear Safety Agency, Yallambie, VIC 3085 Australia

**Keywords:** Injury prevention, Non-ionizing radiation, Health policy, Cosmetic, Consumer protection, Public health

## Abstract

**Background:**

Despite recent regulatory reforms to improve the safety of cosmetic procedures in Australia, treatments involving non-ionizing radiation (NIR)—such as laser, intense pulsed light and radiofrequency—remain largely unregulated in most states and territories. Recent reviews have concluded that there is a lack of evidence of adverse effects, and insufficient evidence has also been cited as a barrier to regulatory reforms. We sought to characterize adverse effects from cosmetic treatments involving NIR reported in Australian media and to analyse associated regulatory themes.

**Methods:**

We searched for Australian news media disseminated between 2008–2023 reporting adverse effects from cosmetic treatments involving non-ionizing radiation (NIR). Identified case reports were coded and analysed to explore adverse effects and associated regulatory insights.

**Results:**

One hundred unique media reports were identified that described ninety-five cases. One in five involved permanent effects with burns and scarring most frequently reported (sixty-five and fifty-four cases respectively). Reports concerned women more than men (eighty vs eight cases), most commonly following laser (sixty cases) or IPL (twenty-nine cases) treatment and in non-clinical rather than clinical settings (sixty vs eighteen cases). Six practitioners collectively accounted for almost one third of cases. Significant regulatory gaps were identified, including insufficient mechanisms for addressing poor professional practice, and barriers to consumers seeking compensation including minimum injury thresholds and uninsured providers.

**Conclusions:**

Media reports have documented cases of serious and permanent injuries following cosmetic NIR treatments in Australia. Nationally consistent regulations should be considered to ensure standards of care, protect consumers, and reduce barriers to redress.

**Supplementary Information:**

The online version contains supplementary material available at 10.1007/s11673-025-10475-y.

## Introduction

In Australia, cosmetics is a lucrative and growing industry, with consumers estimated to spend more than $1 billion annually on non-invasive cosmetic treatments alone (CPCA 2016a). Cosmetic treatments that use non-ionizing radiation (NIR)—such as lasers, intense pulsed light (IPL), radiofrequency, and ultrasound—are increasingly popular with Australians, owing to their broad range of applications, widespread availability, and relatively low cost (ISAPS 2019; CPCA 2016b). These treatments are used for a range of purposes, including: hair removal or promoting hair growth; skin treatments to reduce signs of ageing such as wrinkles or pigmented or vascular lesions; scar revision; treatment of minor skin conditions; tattoo removal; and body shaping, contouring, or sculpting (ICNIRP 2020; Karipidis et al. 2023). More recently, cosmetic NIR has been promoted for “vaginal rejuvenation” although concerns about the safety and efficacy of this procedure have been raised (Digesu et al. 2019).

The International Commission on Non-Ionizing Radiation Protection (ICNIRP) recommends the regulation of cosmetic NIR devices to reduce associated harms (ICNIRP 2020). However, Australian regulations are limited and inconsistent, except for a nationwide solaria prohibition (Karipidis et al. 2019; Karipidis et al. 2023). In 2015, the Australian Radiation Protection and Nuclear Safety Agency (ARPANSA) undertook an analysis to explore options for regulating cosmetic laser and IPL (ARPANSA 2015). Although stakeholders expressed a high level of support for regulatory reforms, jurisdictional regulators concluded that further data on the health burden was required (Urban et al. 2017). Since this time, technological advances have resulted in proliferation in the range of NIR devices and treatments available in Australia (Karipidis et al. 2023).

### Adverse Effects From Cosmetic NIR

Cosmetic treatments involving NIR achieve their intended aesthetic effects by selectively damaging targeted tissue. Consequently, minor, transient, and self-limiting effects such as erythema, swelling and mild to moderate pain are accepted side effects of many treatments (ICNIRP 2020; Al-Niaimi 2016; Breadon and Barnes 2007). More serious or permanent adverse effects can arise from overexposure, including burns, pigment damage, infection, scarring, nerve damage, and eye injury. An understanding of device properties, and of human physiology—including understanding interactions between NIR and tissue—have been suggested as key competencies for preventing adverse effects due to practitioner error (ICNIRP 2020).

There is a lack of evidence on adverse effects of cosmetic NIR use, with the body of evidence comprising small, uncontrolled, non-randomized studies (ICNIRP 2020; Karipidis et al. 2023). Industry-sponsored case studies predominate the literature, with treatment delivered by highly trained operators or clinicians administering high quality treatment protocols. These studies may not be generalizable to the situation in Australia, where regulations are limited and minimum qualifications or training requirements for cosmetic NIR providers are non-existent in most jurisdictions. Reports of severe injuries in Australia have been documented in the media, raising concerns about the injury potential of these treatments and the need for greater regulatory oversight (Karipidis et al. 2023; Brown et al. 2022).

### Regulation in Australia

Excepting the nationwide prohibition of commercial solaria, the regulation of cosmetic NIR use in Australia is limited and inconsistent, comprising a fragmented network of state and commonwealth legislation (Karipidis et al. 2023). Only Tasmania, Queensland, and Western Australia include regulatory controls for cosmetic NIR use in radiation safety legislation. These laws specify minimum training and qualifications for practitioners and—in Queensland and Western Australia—require medical oversight for treatment, however they only apply to class 4 laser (Qld), laser (WA), or laser and IPL (Tas).

Registered health practitioners who provide cosmetic treatments are regulated under the *Health Practitioner Regulation National Law Act 2009* (the National Law), which also prescribes offences for the unlawful use a protected title (s 113) or holding oneself out as a surgeon (s 115 A), registered health practitioner (s 116), or specialist practitioner (s 118) in the absence of corresponding registration. The National Law also specifies requirements for advertising a regulated health service (s 133), with the National Boards jointly developing guidelines for advertising and social media to further explicate these requirements.

Recently, the application of the National Law to the field of cosmetics has been strengthened, to address regulatory gaps identified during an independent regulatory review (Brown et al. 2022). Reforms introduced on July 1, 2023 include a new protected title of “cosmetic surgeon” and guidelines for medical practitioners who perform cosmetic surgery and procedures, with similar guidelines for nurses and other registered health practitioners effective from September 2, 2025 (AHPRA 2016). These guidelines are legally enforceable, providing extrinsic material that can be referenced by regulators during disciplinary or professional proceedings under the National Law (s 139). However, while these measures strengthen the regulations as applied to registered health practitioners, they do not apply to unregistered cosmetic NIR providers. Furthermore, the requirements for advertising a health service may inadvertently funnel demand towards non-clinical providers, to whom no advertising restrictions apply.

In jurisdictions where the National Code of Conduct for Health Care Workers (“the Code”) has been adopted (Qld, Vic, SA, NSW and WA), there is theoretically a mechanism for regulating cosmetic NIR practitioners as unregistered providers of health services. Under Health Care Complaints legislation, such providers must comply with the Code, and prohibition notices can be issued by the regulator where concerns about public health and safety exist. However, “health service” definitions vary by jurisdiction and may not apply to cosmetic NIR use. For example, in New South Wales “health services” are defined via a prescriptive list of professions or circumstances none of which pertain to cosmetic NIR use (*Health Care Complaints Act 1993* (NSW) s 4). In other jurisdictions, the definitions are insufficiently prescriptive to determine whether cosmetic NIR use is encompassed or not.

In the absence of specific legislation, non-invasive cosmetic NIR treatments are ultimately regulated as consumer services and consequently, there are no restrictions on who can offer treatment. Once designated as a consumer service, there is no legal requirement for businesses to hold insurances for professional indemnity or public liability. Similarly, no regulatory mechanisms exist for curtailing service providers, beyond public warnings issued by consumer bodies—even where serious concerns about public health and safety exist. Similarly, devices used in cosmetic NIR procedures are not deemed medical devices by the Therapeutic Goods Administration (TGA), unless they are promoted or intended for therapeutic use (TGA 2023). Consequently, many cosmetic NIR devices are regulated as consumer goods and can be sold without being required to demonstrate compliance with safety and quality of manufacturing standards.

Consumers injured from cosmetic NIR use may be able to sue the service provider or device manufacturer in tort or for breach of contract or failure to comply with the statutory guarantees under the Australian Consumer Law. Claims in negligence for cosmetic NIR injury are subject to minimum injury thresholds, which vary by jurisdiction and may prescribe a minimum level of impairment (for example, fifteen percent of a most extreme case in NSW and greater than five percent whole-person impairment in VIC) (Luntz et al. 2021). Such thresholds operate to exclude small claims, while damages may also be capped (Luntz et al. 2021).

#### Media Influence and Public Policy in Australia

Media coverage can influence public policy, drawing attention to issues, amplifying concerns within the community, and shaping public opinion (Yanovitzky 2002; Happer and Philo 2013; Yanovitzky and Weber 2018). Indeed, the adoption of a national prohibition on solaria in Australia is widely attributed to an intense media campaign surrounding the death of a young woman from melanoma in 2007 (Sinclair and Makin 2008; Jalleh et al. 2008). More recently in Australia, media coverage of “cosmetic cowboys” was instrumental in bringing concerns about regulation of cosmetic procedures to the fore, prompting an independent regulatory review (Brown et al. 2022).

Insufficient data on the health burden from cosmetic NIR use in Australia has been cited by regulators as a barrier to regulatory reforms (Karipidis et al. 2023; Urban et al. 2017). In the absence of robust and generalizable research data, media reports can provide meaningful insights about those most serious injuries—and the associated regulatory response. Accordingly, the present study sought to identify and characterize adverse effects from cosmetic NIR use described in the Australian media between 2008 and 2023. This is the first in a suite of planned studies to examine injuries from cosmetic NIR use in Australia and provide evidence to inform regulatory recommendations.

## Methods

### Data Sources and Searches

Media databases and websites were searched to identify Australian print or audiovisual media content that:

- was disseminated between January 1, 2008 and September 6, 2023 by Australian news or current affairs media (including print or online news media sources, TV news, or current affairs programmes)
- included at least one case report of injury or adverse effects arising from cosmetic NIR treatment in an Australian setting.

Four media databases were searched to identify eligible media reports: ProQuest Australia New Zealand Newstream, Factiva, Newsbank, and Informit TV News.

Searches used key terms relating to NIR-treatments, cosmetic applications, and injury outcomes. Terms were adapted from a previous literature review on the topic (ICNIRP 2020), supplemented or substituted with lay terms to align with media vernacular (see Online Supplementary Materials).

Concerns have previously been raised about the reliability and comprehensiveness of media databases (Hansen and Paul 2015; Weaver and Bimber 2008). Accordingly, additional searches were undertaken to supplement media database searches. This included advanced google news searches, targeted searching of seventeen major Australian news media websites, and contact with key stakeholders (including peak bodies for dermatology, aesthetics and consumer advocacy) to seek historic media held on file.

### Media Report Data Extraction

Descriptive data on media report characteristics were extracted (see Table 1—Online Supplementary Materials). Duplicate media reports (such as those published across different mastheads or syndicated content) were identified and grouped and a single comprehensive (primary) media record identified for coding.

Case reports were identified within media reports, and assigned a unique identifier based on key characteristics. This enabled identification and collation of duplicate case reports across multiple media sources.

### Case Report Coding and Analysis

Coding rules were developed a priori and documented in a comprehensive codebook. Case reports within eligible media were deductively coded to capture data on concepts of interest. These included consumer, treatment/exposure, practitioner/provider, and injury characteristics. The impact of injury and consumers’ views, experiences, and regulatory insights were also coded (see Tables 2 and 3—Online Supplementary Materials). Inductive coding was also undertaken where novel responses were identified.

Case reports described in primary media records were coded in NVivo 14 by a single coder. A randomly selected subset of case reports (15 per cent of all reports) was coded by a second coder to assess inter-coder reliability using Cohen’s kappa (Cohen 1960).

Coded data were exported to Excel and quality assured and cleansed to address incompatibilities or inconsistencies in coding due to discrepant reporting between -or within- media reports. Quantitative analysis was undertaken in Excel. Qualitative analysis was performed within NVivo 14 for high frequency codes describing consumer experience and regulatory themes.

## Results

### Characteristics of Identified Media

**Fig. 1:**
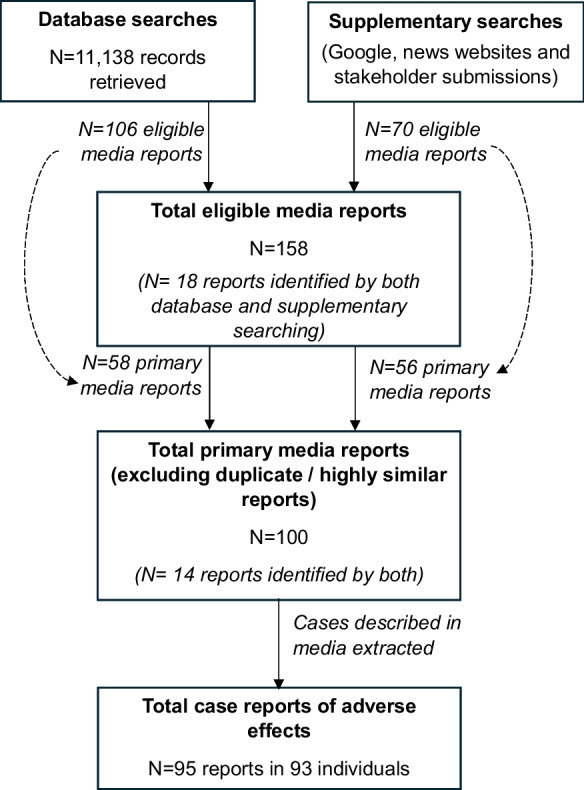
Outcomes of database and supplementary searches

The outcomes of searches and screening are shown at Fig. 1. In total, 158 media reports were identified, comprising one hundred primary media reports (fifty-eight duplicates) in print or audiovisual format (seventy-eight and twenty-two reports respectively). Characteristics of media reports are presented in the Online Supplementary Materials (Table 1).

**Table 1:** Key characteristics of injuries reported in media

Key injury characteristics	N	% all cases	% of cases reporting
***Injured area of body***			
Face (incl. ears, excl. eyes and neck)	42	44%	46%
Arms (incl. underarms, excl. hands and wrists)	15	16%	16%
Legs (excl. feet and ankles)	15	16%	16%
Chest	7	7%	8%
Back	6	6%	7%
Eyes	5	5%	5%
Genitals	5	5%	5%
Hands and wrists	5	5%	5%
Other (incl. stomach, feet and ankles, neck)	6	6%	7%
Injured body area not described	4	4%	N/A
***Acute physical effects* ~ **			
Burns (any)	65	68%	79%
Pain (short-term)	39	41%	48%
Sores or wounds	27	28%	33%
Blistering or vesiculation	24	25%	29%
Redness (erythema)	24	25%	29%
Swelling (oedema)	22	23%	27%
Crusting, scabbing and peeling	16	17%	20%
Inflammation (systemic or localized)	14	15%	17%
Infection	10	11%	12%
Immediate physical effects not described	13	14%	N/A
***Long-term adverse outcomes/injury sequelae* ~ **			
Disfigurement	22	23%	31%
Pigment issues (hypo- or hyper- pigmentation)	28	29%	39%
Scarring	54	57%	76%
UV sensitivity	17	18%	24%
Chronic pain	5	5%	7%
Long term effects/sequelae not described	24	25%	N/A

~ effects reported in < 5 cases not shown here

### Characteristics of Adverse Effects Reported in Media

Included media reports described a total of ninety-five cases of adverse effects from cosmetic NIR use in ninety-three individuals (two people reported adverse outcomes from two separate treatments). Forty-five cases were reported in multiple media reports, including one case documented in twenty-nine media reports.

Case reports varied in the level of detail provided, ranging from a single sentence to comprehensive reports. Most parameters of interest were not routinely detailed in media, with treatment and injury parameters most frequently described, along with gender. Characteristics of case reports are presented in the Online Supplementary Materials (Tables 2 and 3).

#### Consumer, Treatment, and Provider Characteristics

**Fig. 2:**
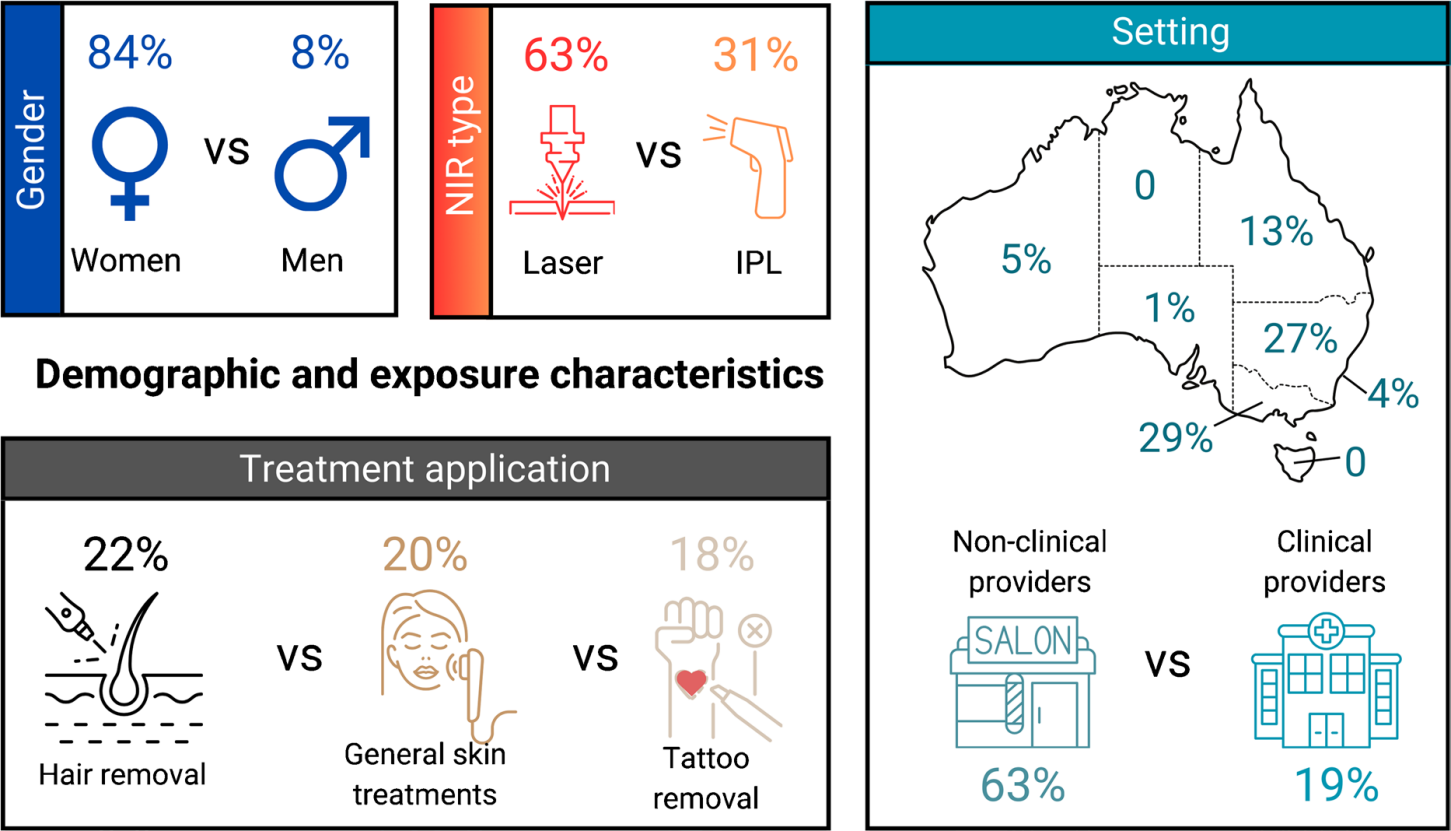
Key demographic and exposure characteristics of injury reports described in media. Data are presented for frequently reported characteristics and do not include all categories. Some reports did not describe parameters of interest, including for gender (8 per cent NR); NIR type (2 per cent); treatment application (19 per cent); treatment location (20 per cent); or provider setting (17 per cent)

Key demographic and exposure characteristics of injury reports are shown in Fig. 2. Those affected ranged in age from sixteen to seventy-three years, with more case reports identified in women than men (eighty vs eight cases). Reports most-commonly involved laser or IPL (sixty and twenty-nine cases respectively) in a non-clinical rather than clinical setting (sixty vs eighteen cases). Treatments for hair removal, general skin enhancement, or tattoo removal accounted for the bulk of case reports (twenty-one, nineteen, and seventeen cases respectively). Six individual practitioners (four non-clinical; two clinical) were associated with multiple reports of injury, collectively accounting for almost one third of cases (twenty-eight cases, collectively).

#### Injuries and Injury Aetiology

Key injury characteristics described in media are presented in Table 1.

Forty-two case reports described causative factors, with the majority attributing injuries to practitioner-related factors (thirty-nine cases), including use of inappropriate treatment protocols or device settings (thirty-three cases), or lack of training or experience (twelve cases). Unsuitable treatment settings were also noted (ten cases), involving inadequate infection control measures or ablative treatments provided in non-clinical settings without requisite clinical care. Links to a selection of media reports, and associated images, are available in the Online Supplementary Materials.

#### Consumer Experience and Impacts

Thirty-six case reports described the consumer’s experience during treatment, including vivid descriptors of pain as being torturous, agony, excruciating, “more painful than childbirth,” or “[so painful that] my husband had to come and hold my feet down” (McKinnon et al. 2011). Cases also consistently described a burning sensation during treatment, likened to being on fire, “cooking from the inside out” (Grimshaw et al. 2013), or like having a match or oxy blowtorch held to the skin. Five women described going into “shock” or “shaking and convulsing” (O’Keefe 2012) during or immediately following treatment. In four cases consumers asked the provider to cease treatment as it was too painful, with this request reportedly ignored in two cases.

Consumers experienced profound psychosocial effects from injuries, with one in five reporting psychological effects and nearly half of all cases reporting quality-of-life impacts (forty-seven cases). These included body image issues (twenty-six cases)—including consistent reports of being unable to look in the mirror—embarrassment or shame (fifteen cases), or feelings of loss, despair, grief or sadness (fourteen cases). Reduced social participation was also prevalent (fourteen cases), with some cases reportedly house-bound for weeks or months, stating: “I feel like a leper” and “It’s ruined my social life” (Bastians 2012).

Financial and work participation impacts were also documented, albeit less frequently (twelve and eleven cases respectively). The cost of injury borne by consumers ranged from $1,000 to $75,000 (median cost $4,000 across five cases). Nine cases reported requiring time off work due to injury—in some cases requiring months of leave—and with two individuals reportedly unable to return to their usual occupations post-injury.

### Legal and Regulatory Themes

#### Legal or Regulatory Matters Reported in Media

Media reports also described various legal and regulatory activities, demonstrating the legislative framework in action. These included tribunal decisions relating to health practitioner misconduct (*Weinstein* [2005] VMPB 3; *Weinstein* [2010] VMPB 7; *The Medical Board of Australia v Al-Naser* [2019] ACAT 52; *Health Ombudsman v Gindi* [2021] QCAT 372), investigations into health practitioner notifications (Thomas and Klan, 2011), and compensation claims for medical negligence (*CDC Clinics v Daemolzekr* [2022] VSCA 54) or a doctor’s failure to warn of risk (*Watson v Kailis* [2008] WADC 95).

In Queensland—where operators of class 4 lasers must be licenced and trained—media described compliance activities conducted in response to reported injuries from laser tattoo removal. Regulators uncovered an unlicenced operator using a class 4 laser incorrectly labelled as a class 3 device, evading the requirement for licencing and training (Kidd 2015).

Despite the limited regulatory controls applied to unregistered providers, a substantial body of legal and regulatory matters pertained to this group of practitioners. These included compensation claims for negligence (*Machado v Advanced Dermatology Group Pty Ltd* [2013] NSWDC 85), prosecution of former doctor—Cynthia Weinstein—and CDC Clinics for holding Ms Weinstein out as a medical practitioner (Australian Health Practitioner Regulation Agency 2016), and prohibition notices issued to Ms Jenny Tran and Mr Andrew Hillery by the NSW Health Care Complaints Commission (NSW HCCC), preventing them from providing health services (NSW HCCC 2014; NSW HCCC 2021). Investigations undertaken by the Victorian Health Care Complaints Commission were also reported (McArthur 2008; Cunningham and Preiss 2019).

#### Consumer Access to Justice

Consumers reported a lack of mechanisms to address poor practice by non-clinical providers: “[Case] had been in contact with government departments, medical boards and health services, but could not stop [provider] from practising” (Polkinghorne 2023, ¶21). Disappointment and dismay at the lack of regulation governing cosmetic procedures was also expressed: “I have become very disheartened with the whole legal process and am not convinced that anything wonderful will come of it because of our laws that say ‘laser falls under the beauty industry’” (Harding 2016, ¶16).

Significant barriers to injury compensation were also described, particularly for treatments provided in non-clinical settings. In three cases, claiming compensation was deemed unfeasible because the non-clinical provider was uninsured. This also made accessing legal representation difficult: “it turns out [the provider] wasn’t insured, so legal representatives won’t take us on as a no-win, no-fee, and our problem is we can’t fund out of our own pockets” (Polkinghorne 2023, ¶23). Consumers also reported that—despite their injuries being significant and distressing—they did not meet the minimum thresholds for compensation: “Because my injuries didn’t result in permanent damage, I didn’t have a case” (Callander 2017, ¶3). In one case, a consumer awarded $230,000 in compensation reported being unable to obtain payment because the practitioner—Ms Jenny Tran—declared bankruptcy, although she allegedly continued to treat clients at a new business owned by her spouse (Grimshaw and Piotrowski 2013). Media reports attributed a further three cases of facial injuries to Ms Tran’s ongoing practice (O’Keefe 2012).

## Discussion

### Key Findings on Adverse Effects

We documented ninety-five cases of adverse effects arising from cosmetic NIR treatments in Australia, reported in the media between 2008 and 2023. Case reports described a range of adverse effects including burns, scarring, and pigment damage, and documented significant psychosocial and financial impacts. These findings indicate that serious, permanent, and long-lasting injuries are occurring following cosmetic NIR use in Australia. Our analysis also suggests an inequitable burden of injury, with a preponderance of cases reported in women.

Practitioner skill, training, and experience has been suggested as a key aetiologic factor underlying preventable injury (Karipidis et al. 2023; Hammes et al. 2013). With six practitioners (predominantly non-clinical) collectively accounting for almost one third of cases, it is evident that NIR devices have significant injury potential in the hands of less skilled or reckless practitioners. Furthermore, practitioner-related factors were reported in ninety-three percent of cases that described a causative factor (thirty-nine cases in total), and a greater number of cases were reported in non-clinical settings. This further suggests a link between practitioner knowledge and injury burden. Further research is required to understand the relationship between consumer, treatment, and provider factors, and the risk of adverse effects in an Australian context.

### Regulatory Implications

Our analysis provides critical regulatory insights, suggesting that the current legal framework lacks adequate mechanisms to respond to poor practice and provide consumer redress—particularly in non-clinical settings. Systemic barriers to accessing compensation for personal injury were documented in media reports, including the operation of minimum injury thresholds and the uninsured status of some non-clinical providers. Consumers reported that these factors impacted legal services access, with conditional “No Win, No Fee” arrangements unavailable due to the perceived low prospect of claim success.

Despite the self-reported nature of these regulatory concerns, they align with broader themes from research on consumers’ experiences with legal services in Australia. Studies suggest that unmet legal needs are prevalent among people who have experienced personal injury, with drivers including lack of individual capacity or perceptions that the process would be too lengthy, stressful, or costly (Coumarelos et al. 2017; Balmer et al. 2023). Given the role of costs in driving consumer decisions about legal services access, the inability to access conditional fee arrangements logically presents a significant barrier to consumer redress.

It has been noted that personal injury lawyers exercise significant choice in client selection (Grant 2021) and that firms that offer conditional arrangements may be unlikely to accept high risk cases or those where damages awarded may be low (Law Council of Australia 2021). This aligns with consumer assertions that in the presence of an uninsured NIR treatment provider, lawyers were unwilling to act for the injured person on a “No Win, No Fee” basis—likely due to the limited prospect of recovering compensation. The failure of unregistered providers to hold appropriate indemnity insurance has been documented in regulatory proceedings (NSW HCCC 2021), lending weight to consumers’ experiences in this regard.

Consumers also reported that minimum thresholds of injury presented a significant barrier to achieving redress. Except for the most egregious cases, injuries from cosmetic NIR treatments are likely to affect discrete regions of the body and may lessen in appearance or resolve over time. Such injuries may not meet the minimum thresholds for actionable harm. Indeed, in its 2014 inquiry into the Wrongs Act the Victorian Competition and Efficiency Commission reported difficulties satisfying minimum thresholds for injuries involving permanent scarring (VCEC 2014). Inquiry participants also recommended that burn and scar injuries should be excepted from minimum threshold requirements, to address barriers to accessing compensation for non-economic loss in these circumstances.

The exclusion of secondary psychiatric or psychological impairments from calculations of injury severity may also impede compensation access relating to cosmetic NIR injuries. Our media analysis found that the psychosocial impacts of injuries from cosmetic NIR can be significant and life-altering. However, the law of negligence deems these experiences to be inconsequential losses borne by injured persons, rather than actionable harms. This disregards the inextricable link between physical appearance and psychological well-being for many individuals who seek cosmetic treatments, raising further questions about the normative value of severity thresholds where a significant element of harm is excluded when calculating a threshold of impairment. The consumer experiences reported in media echo broader criticisms of the tort law reforms enacted in the early 2000s. These include that the thresholds imposed lack normative justification, that they lead to disparities and inequities and that they have shifted the burden of costs away from negligent tortfeasors and insurers on to consumers (Field 2008; Priaulx 2012; Kerridge et al. 2013).

Finally, because cosmetic NIR is regulated as a “consumer” rather than “health or medical” service, in most jurisdictions there are no regulations that specify minimum safety and quality standards for these services. Our analysis identified reports of high-risk ablative procedures being performed in non-clinical settings that lack both suitable infection control procedures and access to medication such as antibiotics and anaesthesia. These provisions are vital to ensure safe, comfortable, and effective treatment for consumers, yet they are seemingly not assured under current regulations.

Our analysis also identified reports of the regulatory system in action, including matters relating to regulation of registered and unregistered health practitioners, and compensation claims. Collectively, these records highlight the numerous and varied levers available to regulators in relation to cosmetic NIR. However, far from providing assurance that robust legal and regulatory mechanisms exist to adequately address poor professional practice and protect consumers, these sources highlight the specificity of regulatory controls and challenges in enforcement.

First, whilst the cases of Ms Tran and Mr Hillery may suggest that prohibition notices can be issued to cosmetic NIR providers for breaches of the Code, the reality is more complex. Specific factors in these matters contributed to the conclusion that the individuals were providing “health services” under the *Health Care Complaints Act 1993* (NSW). They involved the unauthorized supply of prescription pharmaceuticals (NSW HCCC 2014; NSW HCCC 2021) and misrepresentation of a person as a health practitioner (NSW HCCC 2021). These seem to be the dominant features that enabled prohibition notices to be issued, given that the definition of “health service” under s 4 of the *Health Care Complaints Act 1993* (NSW) does not encompass cosmetic NIR treatments. In the absence of these kinds of additional factors, cosmetic NIR is unlikely to meet the legal definition of a “health service” and therefore escapes regulatory coverage under this mechanism. Even where prohibition notices are issued in such circumstances, they may not prevent providers from continuing to offer or perform cosmetic NIR treatments, as the prohibition applies only to the provision of “health services.”

The impact of these regulatory shortcomings are highlighted in the case of Ms Jenny Tran. Although a prohibition notice was ultimately issued to Ms Tran (NSW HCCC 2014), the notice was issued more than four years after the initial serious injury occurred and did not pertain to cosmetic NIR use. In the intervening period, media reports attributed three more cases of injury to Ms Tran’s ongoing practice (O’Keefe 2012).

Similarly, in the case of Cynthia Weinstein and CDC clinics, these parties were the subject of numerous occupational tribunal and civil proceedings spanning a twenty-year period during which regulators gradually restricted Ms Weinstein’s practice. Far from evidencing effective regulatory controls in action, this circumstance suggests that regulatory controls are sluggish and ineffective, favouring a practitioner’s right to practice over consumer safety. These findings are echoed by media reporting on a NSW HCCC investigation into Dr Adrianna Scheibner, which suggested that the Medical Board was slow to act despite safety concerns raised both by consumers and Dr Scheibner’s colleagues (Thomas and Klan 2011).

Finally, two practitioners were associated with injuries in more than one jurisdiction, highlighting the need for a nationally consistent approach to prevent practitioners from relocating their practice to circumvent regulatory consequences. Indeed, in its Statement of Decision regarding Ms Jenny Tran, the NSW HCC noted suspicions that she had relocated and commenced operation in Queensland to “open a new shop” (NSW HCCC 2014). Under corresponding Health Care Complaints legislation, a prohibition notice issued in NSW also takes effect in Queensland, however it is unclear whether attempts were made to enforce the prohibition notice inter-jurisdictionally.

Whilst a comprehensive analysis of the regulations was beyond the scope of this study, cases reported in media suggest that significant regulatory gaps—and enforcement challenges—exist. The regulatory complexity in this area necessitates further examination of regulatory data before specific reforms can be proposed. Any such analysis should explore a broad range of data sources to elucidate how current legislation is being applied, and the outcomes being delivered for individual consumers and from a public health perspective more generally. This will aid in fully characterizing regulatory gaps and may identify existing mechanisms that can be adopted more broadly to provide a nationally consistent regulatory framework. At a minimum, any such framework must provide appropriate avenues for consumer redress, promote standards of care that ensure safety, quality and ethical practice aligned with community expectations, and provide avenues for regulatory intervention in the face of poor provider practice. Regulatory reforms alone are insufficient, unless accompanied by appropriate enforcement activities—with inter-jurisdictional co-operation—to foster a culture of compliance within the sector.

### Strengths and Limitations

Our study sought to comprehensively characterize case reports described in media and included supplementary searches to address concerns about the reliability of media databases (Hansen and Paul 2015; Weaver and Bimber 2008). Methods for coding were also well planned and documented, utilizing a consistent coding frame across media reports, as demonstrated by the high level of inter-coder agreement (Cohen’s kappa = 0.69; Cohen, 1960).

Despite our rigorous searches involving multiple databases and comprehensive search terms, one third of eligible reports were not identified via database searches (see Fig. 1). This suggests that significant gaps exist in media database coverage, and consequently our analysis has likely not identified all in-scope media. Our experience accords with previous studies which described significant deficiencies in media database coverage (Hansen and Paul 2015; Weaver and Bimber 2008). This finding has significant methodological implications for future media analyses in Australia.

The main limitation of our study is that it presents media-derived case reports, which are per definition selected based on newsworthiness or relevance to a particular narrative rather than being a systematically collected, representative sample. Accordingly, injuries with particular features may be favourably reported over others or facts selectively reported to suit the media narrative. Missing data due to incomprehensive or unclear reporting was also prevalent, with most parameters of interest not consistently or comprehensively described. Furthermore, parameters were generally self-reported by consumers or journalists and may be inaccurate or unverified.

Media interest in a topic varies by region and over time. Accordingly, fluctuations in reported injuries reflect changes in temporal and regional media contexts and do not permit inferences about the prevalence of injury over time or by jurisdiction. Finally, while it presents a snapshot of those more serious injuries that have occurred, this analysis does not facilitate quantification of the burden of injury from cosmetic NIR use in Australia. This is a critical knowledge gap that must be explored, to inform regulatory decision-making.

## Conclusions

Despite sustained growth in cosmetic NIR use in Australia over the previous decade, adverse effects associated with these treatments remain poorly understood. Our study suggests that serious, preventable injuries are occurring following cosmetic NIR treatments, and that these injuries can have profound, life-altering, and long-lasting impacts.

Significant barriers exist that prevent effective self-regulation and consumer redress, particularly where injuries are acquired in a non-clinical setting. Regulatory reforms to achieve nationally consistent regulations that establish standards of care, ensure public safety, and reduce barriers to redress for injuries are long overdue. Any regulatory change must be accompanied by routine enforcement activities, to build a culture of compliance within the sector.

These findings are a critical first step towards better understanding injuries from cosmetic use of NIR in Australia. Further research to better understand the current regulations, explore regulatory approaches and to characterize the burden of injury, aetiology, and associated impacts is required, to inform regulatory recommendations.

## Supplementary Information

Below is the link to the electronic supplementary material.

Supplementary file1 (DOCX 83 KB)

## Data Availability

Data are available on reasonable request.
