# Supplementary material for: Burned in Pursuit of Beauty: Injuries From Cosmetic Use of Non-Ionizing Radiation and Associated Regulatory Gaps
Source: J Bioeth Inq. 2025 Sep 24;23(2):327–39. doi: 10.1007/s11673-025-10475-y (PMC13388330; doi:10.1007/s11673-025-10475-y)
Supplement: Supplementary file 1 — Supplementary file1 (DOCX 83 KB) [file 11673_2025_10475_MOESM1_ESM.docx]

# **Supplementary materials**

## **Database searches**

News media databases (Newsbank, Factiva, ProQuest Australia & New Zealand Newsstream and Informit TVNews) were searched using key terms and filters, to identify relevant Australian media content.

Database searching combined key terms relating to NIR-treatments, cosmetic applications, and injury outcomes to identify relevant media articles. Key terms were adapted from those developed for use in a review conducted by the International Commission for Non-ionising Radiation Protection (ICNIRP), supplemented or substituted with lay terms to reflect the language used in media reports.

Key terms included:

***NIR treatment terms:*** *laser, light treatment, light therapy, IPL, intense pulsed light, LED, electromagnetic, EMF, radiofrequency, RF, microwave, ultrasound, high-intensity focused ultrasound, HIFU, microfocused ultrasound, MFU.*

***Cosmetic applications:*** *cosmetic, beauty, aesthetic, esthetic, epilation, hair, tattoo, skin, rejuvenation, wrinkle, scar, vagina, vaginal, acne, rosacea, pigment, pigmentation, lipolysis, lipo, fat, cellulite, body, vascular, vein, brazilian, brasilian, bikini, capillary, capillaries*

***Injury terms or adverse outcomes:*** *injury, injured, injuries, botch, botched, harm, harmful, pain, painful, impaired, impairment, discomfort, abscess,* *burn, burns, burned, scar, scarred, scarring, nerve, blind, blinded, blinding, eye, visual, vision, pupil, iris, cornea, blister, blistering, blistered, crust, crusted, crusty, crusting, bruise, bruised, bruising, lump, nodule, abscess, swelling, swollen, allergy, allergic, blood, bleeding, inflamed, inflammation*

Exposure, injury and treatment terms were combined with Boolean operators and database-specific syntax to develop search strategies specific to each database. Terms were also truncated with use of wild card operators where appropriate, to optimise searches.

Filters were applied to search results to restrict results to Australian news media content published after 1 January 2008.

Targeted searching of Meltwater - the Australian Radiation Protection and Nuclear Safety Agency’s media aggregator tool - was also undertaken, although at that time the tool did not allow historical searches beyond 12 months and did not yield any relevant material.

Table 1 - Characteristics of media reports

|  | **All (including duplicates)** | **Unique (primary) reports** |
| --- | --- | --- |
| *Media source jurisdiction:* | | |
| ACT | 2 | 2 |
| NT | 2 | 1 |
| NSW | 37 | 8 |
| Qld | 14 | 8 |
| SA | 1 | 0 |
| Tas | 1 | 0 |
| VIC | 27 | 16 |
| WA | 11 | 7 |
| National | 63 | 58 |
| *Media type:* | | |
| Newspaper (including online sites of same) | 99 | 45 |
| Newswire | 2 | 2 |
| Online news website | 34 | 30 |
| Online newsletter (linked via news website) | 1 | 1 |
| TV news | 5 | 5 |
| TV current affairs program | 17 | 17 |
| *Number of case reports described by media*:* | | |
| 1 | 114 | 63 |
| 2 | 24 | 20 |
| 3 or more | 20 | 17 |
| **Total** | **158** | **100** |

*some case reports (N=45, 47%) were described in >1 media report

Table 2 – Number and percentage of case reports that documented parameters of interest

| **Parameter of interest** | **N**  **(Total N=95)** | **% of Total** |
| --- | --- | --- |
| **Consumer characteristics** | | |
| *Age at time of treatment* | 31 | 33% |
| *Sex* | 88 | 93% |
| *Consumer risk factors or contraindications* | 17 | 18% |
| **Treatment characteristics** | | |
| *Type of NIR used in treatment* | 93 | 98% |
| *Treatment purpose / intent* | 77 | 81% |
| *Treatment setting (clinical vs non-clinical)* | 79 | 83% |
| *Year of treatment* | 50 | 53% |
| **Provider characteristics** | | |
| *Practitioner qualifications* | 53 | 56% |
| *Provider location* | 76 | 80% |
| **Consumer views & experience** | | |
| *Understanding of risk pre-treatment* | 14 | 15% |
| *Experience during treatment* | 36 | 38% |
| *Adequacy of provider care* | 46 | 48% |
| **Injuries & adverse effect characteristics** | | |
| *Injured area of body* | 91 | 96% |
| *Injuries and adverse outcomes – immediate physical effects* | 82 | 86% |
| *Injuries and adverse outcomes – long-term outcomes & sequelae* | 71 | 75% |
| *Medical treatment – immediately post-injury* | 38 | 40% |
| *Medical treatment – long-term effects, sequelae & restore appearance* | 21 | 22% |
| *Stated factors contributing to injury* | 42 | 44% |
| *Time to recovery* | 40 | 42% |
| **Impacts of injury** | | |
| *Financial impacts* | 12 | 13% |
| *Psychological impacts* | 19 | 20% |
| *Quality of life impacts* | 47 | 49% |
| *Work participation impacts* | 11 | 12% |
| **Consumer reporting action** | | |
| *Reporting action taken by consumer in response to injury* | 29 | 31% |
| *Consumer- reported barriers to regulatory action* | 11 | 12% |

Table 3- Characteristics of media-derived case reports

| **Parameters** | **N** | **% (all)** | **% (where data available)** |
| --- | --- | --- | --- |
| ***Consumer characteristics*** | | | |
| ***Gender:*** | | | |
| Female | 80 | 84% | 91% |
| Male | 8 | 8% | 9% |
| Non-binary / Other | 0 | 0% | 0% |
| Gender not reported / discernible | 7 | 7% | NA |
| ***Age:*** | | | |
| 19 years or younger | 1 | 1% | 3% |
| 20-29 years | 9 | 9% | 29% |
| 30-39 years | 7 | 7% | 23% |
| 40-49 years | 8 | 8% | 26% |
| 50-59 years | 5 | 5% | 16% |
| 60-69 years | 0 | 0% | 0% |
| 70-79 years | 1 | 1% | 3% |
| 80+ years | 0 | 0% | 0% |
| Age not reported / discernible | 64 | 67% | N/A |
| **Total** | **95** | **100%** | **100%** |
| ***Consumer risk factors:*** | | | |
| ***Natural skin tone:*** |  |  |  |
| Darker skinned (Fitzpatrick Type IV-VI) | 4 | 4% | 31% |
| Lighter skinned (Fitzpatrick Type I-III) | 9 | 9% | 69% |
| Skin tone not reported / discernible | 82 | 86% | N/A |
| **Total** | **95** | **100%** | **100%** |
| ***Other risk factors / contraindications reported:*** |  |  |  |
| Heavy tan or high UV exposure pre or post-treatment | 1 | 1% | N/A |
| Use of photosensitising medications or supplements | 0 | 0% | N/A |
| Pre-existing medical conditions (including skin conditions, medical implants) | 3 | 3% | N/A |
| ***Treatment / exposure characteristics*** |  |  |  |
| ***Type of NIR:*** | | | |
| Combination NIR | 2 | 2% | 2% |
| Radiofrequency (RF) | 2 | 2% | 2% |
| Optical / light (not otherwise specified) | 2 | 2% | 2% |
| Intense pulsed light (IPL) | 29 | 31% | 31% |
| Laser | 60 | 63% | 65% |
| High-intensity focused ultrasound (HIFU) | 2 | 2% | 2% |
| NIR type not reported / discernible | 2 | 2% | N/A |
| **Total** | **99^^^** | **104%^^^** | **104%^^^** |
| ***Treatment intent:*** | | | |
| Hair removal or epilation | 21 | 22% | 27% |
| Skin treatments (various, excluding tattoo removal) | 40 | 42% | 52% |
| *Skin treatment (not further specified)* | *1* | *1%* | *1%* |
| *Eyebag or eyelid treatment* | *3* | *3%* | *4%* |
| *General skin enhancement* | *19* | *20%* | *25%* |
| *Pigmentation* | *11* | *12%* | *14%* |
| *Scar reduction* | *6* | *6%* | *8%* |
| *Skin conditions* | *2* | *2%* | *3%* |
| *Vascular lesions* | *4* | *4%* | *5%* |
| *Wrinkle reduction* | *8* | *8%* | *10%* |
| Tattoo removal | 17 | 18% | 22% |
| Treatment purpose not reported / discernible | 18 | 19% | N/A |
| **Total** | **96*** | **101%*** | **101%*** |
| ***Year of treatment*** | | | |
| Pre-2007 | 8 | 8% | 16% |
| 2007 | 2 | 2% | 4% |
| 2008 | 0 | 0% | 0% |
| 2009 | 2 | 2% | 4% |
| 2010 | 3 | 3% | 6% |
| 2011 | 3 | 3% | 6% |
| 2012 | 6 | 6% | 12% |
| 2013 | 0 | 0% | 0% |
| 2014 | 3 | 3% | 6% |
| 2015 | 2 | 2% | 4% |
| 2016 | 7 | 7% | 14% |
| 2017 | 2 | 2% | 4% |
| 2018 | 3 | 3% | 6% |
| 2019 | 1 | 1% | 2% |
| 2020 | 5 | 5% | 10% |
| 2021 | 0 | 0% | 0% |
| 2022 | 2 | 2% | 4% |
| 2023 | 1 | 1% | 2% |
| Year not reported / discernible | 45 | 47% | N/A |
| **Total** | **95** | **100%** | **100%** |
| ***Treatment setting*** | | | |
| Clinical | 18 | 19% | 23% |
| Non-clinical | 60 | 63% | 76% |
| Home-use | 1 | 1% | 1% |
| Setting not reported / discernible | 16 | 17% | N/A |
| **Total** | **95** | **100%** | **100%** |
| ***Practitioner / provider characteristics*** |  |  |  |
| ***Provider location*** | | | |
| ACT | 4 | 4% | 5% |
| NSW | 26 | 27% | 34% |
| NT | 0 | 0% | 0% |
| QLD | 12 | 13% | 16% |
| SA | 1 | 1% | 1% |
| TAS | 0 | 0% | 0% |
| VIC | 28 | 29% | 37% |
| WA | 5 | 5% | 7% |
| Location not reported / discernible | 19 | 20% | N/A |
| **Total** | **95** | **100%** | **100%** |
| ***Practitioner qualifications*** | | | |
| Beauty therapy, or related qualification | 24 | 25% | 45% |
| Medical practitioner | 14 | 15% | 26% |
| NIR-specific or device-specific qualification | 15 | 16% | 28% |
| No qualifications | 7 | 7% | 13% |
| Other health professional (eg. nurse) | 2 | 2% | 4% |
| Other related qualifications (eg. dermoscopy) | 7 | 7% | 13% |
| Self-administered | 0 | 0% | 0% |
| Practitioner qualifications not reported / discernible | 42 | 44% | N/A |
| **Total** | **111^#^** | **117%^#^** | **130%^#^** |
| ***Injury / adverse effect characteristics*** |  |  |  |
| ***Injured area of body*** | | | |
| Arms (including underarms) | 15 | 16% | 16% |
| Hands & wrists | 5 | 5% | 5% |
| Back | 6 | 6% | 7% |
| Chest | 7 | 7% | 8% |
| Shoulders | 0 | 0% | 0% |
| Stomach | 1 | 1% | 1% |
| Eyes | 5 | 5% | 5% |
| Face (including ears, excluding eyes and neck) | 42 | 44% | 46% |
| Genitals | 5 | 5% | 5% |
| Legs | 15 | 16% | 16% |
| Feet & ankles | 1 | 1% | 1% |
| Neck | 4 | 4% | 4% |
| Injured body area not reported / discernible | 4 | 4% | N/A |
| **Total** | **110~** | **116%~** | **116%~** |
| ***Immediate physical effects*** | | | |
| Bruising | 4 | 4% | 5% |
| Swelling (oedema) | 22 | 23% | 27% |
| Inflammation (systemic or localised) | 14 | 15% | 17% |
| Lethargy or fatigue | 1 | 1% | 1% |
| Allergic reaction | 2 | 2% | 2% |
| Infection | 10 | 11% | 12% |
| Altered sentation (tingling, numbness, sensitivity) - short term | 2 | 2% | 2% |
| Headache | 1 | 1% | 1% |
| Pain (short-term) | 39 | 41% | 48% |
| Burns (any) | 65 | 68% | 79% |
| Blistering or vesiculation | 24 | 25% | 29% |
| Crusting, scabbing and peeling | 16 | 17% | 20% |
| Itchy skin (pruritus) | 2 | 2% | 2% |
| Redness (erythema) | 24 | 25% | 29% |
| Acne or related lesions | 4 | 4% | 5% |
| Purple rash (purpura) | 1 | 1% | 1% |
| Small red dots and bumps (Petechia) | 1 | 1% | 1% |
| Skin thickening and hardening (induration) | 1 | 1% | 1% |
| Sores or wounds | 27 | 28% | 33% |
| Ulcers | 1 | 1% | 1% |
| Eye injury | 4 | 4% | 5% |
| Increased hair growth | 1 | 1% | 1% |
| Oxidised tattoo ink | 1 | 1% | 1% |
| Immediate physical effects not reported / discernible | 13 | 14% | N/A |
| ***Long-term adverse outcomes / injury sequelae*** | | | |
| Acne or related lesions | 4 | 4% | 5% |
| Sensitive or delicate skin | 3 | 3% | 4% |
| Chronic pain | 5 | 5% | 7% |
| Headache (long-term) | 1 | 1% | 1% |
| Ongoing nerve damage | 1 | 1% | 1% |
| Disfigurement | 22 | 23% | 31% |
| Pigment issues (hypo- or hyper- pigmentation) | 28 | 29% | 39% |
| Scarring | 54 | 57% | 76% |
| UV sensitivity | 17 | 18% | 24% |
| Long term effects / sequelae not reported / discernible | 24 | 25% | N/A |
| ***Consumer experience/symptoms during treatment*** | | | |
| Bleeding | 5 | 5% | 14% |
| Distress | 3 | 3% | 8% |
| Heat or burning sensation | 16 | 17% | 44% |
| Pain | 31 | 33% | 86% |
| Requested to stop treatment | 4 | 4% | 11% |
| Trauma or shock (incl shaking) | 5 | 5% | 14% |
| Unpleasant smell | 4 | 4% | 11% |
| Treatment experience not reported / discernible | 59 | 62% | N/A |
| ***Time to recovery*** | | | |
| 2 weeks to 3 months | 1 | 1% | 3% |
| 3 to 6 months | 1 | 1% | 3% |
| 6 to12 months | 3 | 3% | 8% |
| 7 days or less | 0 | 0% | 0% |
| 7 days to 2 weeks | 0 | 0% | 0% |
| more than 12 months | 0 | 0% | 0% |
| Permanent effects | 21 | 22% | 53% |
| Recovery ongoing | 16 | 17% | 40% |
| Time to recovery not reported / discernible | 55 | 58% | N/A |
| ***Medical treatment required immediately post injury*** | | | |
| Hospital presentation or admission | 8 | 8% | 21% |
| Pharmaceutical & other interventions or procedures | 19 | 20% | 50% |
| Surgical procedures | 0 | 0% | 0% |
| Treatment by medical professional | 32 | 34% | 84% |
| Treatment by other health professional | 2 | 2% | 5% |
| Acute medical treatment not reported / discernible | 57 | 60% | N/A |
| ***Medical treatment required to treat long-term effects & restore appearance*** | | | |
| Pharmaceutical & other interventions or procedures | 10 | 11% | 48% |
| Surgical procedures | 4 | 4% | 19% |
| Treatment by medical professional | 15 | 16% | 71% |
| Treatment by mental health professional | 3 | 3% | 14% |
| Long-term medical treatment not reported / discernible | 74 | 78% | N/A |
| ***Reported aetiologic factors*** | | | |
| Consumer-related factors (any/all) | 3 | 3% | 7% |
| *Consumer compliance with aftercare* | *2* | *2%* | *5%* |
| *Consumer risk factors* | *1* | *1%* | *2%* |
| Device-related factors (any/all) | 2 | 2% | 5% |
| *Device quality* | *1* | *1%* | *2%* |
| *Poorly maintained devices* | *1* | *1%* | *2%* |
| Practitioner-related factors (any/all) | 39 | 41% | 93% |
| *Inadequate protective measures (including eye protection and cooling measures)* | *3* | *3%* | *7%* |
| *Inappropriate treatment protocol (including inappropriate device for purpose, inappropriate device settings)* | *33* | *35%* | *79%* |
| *Inadequate standards of care (intake procedures, after care advice)* | *5* | *5%* | *12%* |
| *Lack of training or experience* | *12* | *13%* | *29%* |
| Unsuitable setting (all) | 10 | 11% | 24% |
| *Unsanitary environment & poor infection control* | *2* | *2%* | *5%* |
| *Unsuitable setting (should be performed in clinical setting only)* | *8* | *8%* | *19%* |
| Factors contributing to injury not reported / discernible | 53 | 56% | N/A |
| ***Impacts of injury (financial and psychosocial)*** | | | |
| ***Financial impacts*** | | | |
| Financial impacts (not otherwise specified) | 7 | 7% | 58% |
| Legal costs | 2 | 2% | 17% |
| Lost income | 1 | 1% | 8% |
| Medical costs | 7 | 7% | 58% |
| Financial impacts not reported / discernible | 83 | 87% | N/A |
| ***Psychological impacts*** | | | |
| Poor mental health (not otherwise described) | 4 | 4% | 21% |
| Anxiety | 10 | 11% | 53% |
| Depression | 6 | 6% | 32% |
| Trauma or PTSD | 3 | 3% | 16% |
| Psychological impacts not reported / discernible | 76 | 80% | N/A |
| ***Quality of Life impacts*** | | | |
| Quality of life impacts (not further specified) | 3 | 3% | 6% |
| Increased negative emotions (not further specified) | 1 | 1% | 2% |
| Anger or rage | 6 | 6% | 13% |
| Despair, grief, sadness or sense of loss | 14 | 15% | 30% |
| Embarrassment or shame | 15 | 16% | 32% |
| Guilt or self-blame | 6 | 6% | 13% |
| Regret | 8 | 8% | 17% |
| Reduced physical health | 7 | 7% | 15% |
| Poor or reduced sleep | 6 | 6% | 13% |
| Reduced positive emotions (reduced hope, mood, joy and pleasure) | 3 | 3% | 6% |
| Reduced social participation | 14 | 15% | 30% |
| Reduced wellbeing, life meaning and satisfaction | 12 | 13% | 26% |
| Impacts on sexuality / sexual function | 1 | 1% | 2% |
| Reduced body-image | 26 | 27% | 55% |
| Reduced self-image | 10 | 11% | 21% |
| Quality of life impacts not reported / discernible | 48 | 51% | N/A |
| ***Work participation*** | | | |
| Reduced work participation (other / not-specified) | 5 | 5% | 45% |
| Reduced income potential due to injury or adverse effects | 0 | 0% | 0% |
| Time off work due to injury or recovery | 9 | 9% | 82% |
| Work participation impacts not reported / discernible | 84 | 88% | N/A |
| ***Consumer views, experience and actions*** | | | |
| ***Consumer views about understanding of risks pre-treatment*** | | | |
| No- or limited- understanding of risk pre-treatment | 13 | 14% | 93% |
| Some- or good- understanding of risk pre-treatment | 1 | 1% | 7% |
| ***Consumer views about adequacy of provider care*** | | | |
| Adequate treatment aftercare advice | 0 | 0% | 0% |
| No- or inadequate- treatment aftercare advice | 0 | 0% | 0% |
| Adequate informed consent process | 0 | 0% | 0% |
| No- or inadequate- informed consent process | 19 | 20% | 23% |
| Adequate intake process | 0 | 0% | 0% |
| No- or inadequate- intake process | 1 | 1% | 1% |
| Good or adequate response to injury | 1 | 1% | 1% |
| Poor or inadequate response to injury (all/various reasons) | 34 | 36% | 36% |
| *Poor or inadequate response to injury (not otherwise specified)* | *15* | *16%* | *16%* |
| *Denied injury had occurred* | *20* | *21%* | *22%* |
| *Denied responsibility for injury or adverse outcome* | *10* | *11%* | *19%* |
| *Inappropriate treatment recommendations* | *12* | *13%* | *13%* |
| *Non-responsive to contact* | *3* | *3%* | *4%* |
| Adequacy of provider care not reported / discernible | 49 | 52% | N/A |
| ***Consumer regulatory reporting action*** | | | |
| Consumer complaint | 2 | 2% | 7% |
| Cosmetic therapy peak body or professional association | 0 | 0% | 0% |
| Government (other) | 3 | 3% | 10% |
| Health care complaint | 9 | 9% | 31% |
| Legal action | 13 | 14% | 45% |
| No action | 8 | 8% | 28% |
| Parliamentary member | 0 | 0% | 0% |
| Therapeutic Goods Administration | 0 | 0% | 0% |
| Consumer regulatory action not reported / discernible | 66 | 69% | N/A |
| ***Consumer reported barriers to regulatory action*** | | | |
| Legal action unfeasible | 3 | 3% | 27% |
| Not confident would be believed | 2 | 2% | 18% |
| Report unlikely to result in action or affect change | 0 | 0% | 0% |
| Signed a legal waiver | 3 | 3% | 27% |
| Too embarrassed | 1 | 1% | 9% |
| Unaware had suffered an injury or adverse effect | 2 | 2% | 18% |
| Unsure where to direct complaint | 1 | 1% | 9% |
| Regulatory barriers not discussed / reported | 84 | 88% | N/A |

^ two cases were combined treatments that included two types of NIR; * one case reported a dual-purpose treatment to remove hair and rejuvenate skin on the face; ^#^ some practitioners held more than one qualification; ~ some cases involved injury to >1 body part

Table 4- Links to a selection of media reports inclusive of images of injuries

| **Description** | **Link** |
| --- | --- |
| Two cases of burns to hands from tattoo removal, Qld | <https://www.news.com.au/lifestyle/beauty/face-body/tattoo-laser-removal-queensland-man-suffers-serious-burns-after-trying-to-get-his-tattoos-removed/news-story/183200c8ca225d4df1d2f82dc8a40696> |
|  | <https://www.couriermail.com.au/news/queensland/bundaberg/laser-tattoo-removal-leaves-20yearold-with-severe-burns/news-story/2cd23a34de06b6168f909d20c8d9159c> |
| Burns to legs following IPL hair removal, Qld | <https://www.abc.net.au/news/2021-09-17/ipl-regulation-calls-burns-allegations/100445636> |
| Three cases of burns to face from laser skin rejuvenation, VIC | <https://au.news.yahoo.com/when-beauty-treatments-turn-ugly-37091109.html> |
| Two cases of burns from hair removal, VIC | <https://www.9news.com.au/national/victorian-woman-suffers-extensive-second-degree-burns-in-botched-laser-procedure-geelong/3f04ba76-653e-458c-87a0-d0d75132> |
| Burns following laser skin rejuvenation, ACT | <https://www.abc.net.au/news/2019-09-08/cosmetic-laser-treatments-need-tighter-regulation-patients-warn/11489196> |
| Four cases of burns to the face and underarms following various treatments, Vic (N=3) or unspecified (N=1) | <https://www.9news.com.au/national/a-current-affair-laser-beauty-treatments-burns-claims-women-latest-news-australia/895572cf-ad64-48b6-9028-09db43b34fc2> |
